# Boosting the Immune System for HIV Cure: A γδ T Cell Perspective

**DOI:** 10.3389/fcimb.2020.00221

**Published:** 2020-05-19

**Authors:** Brendan T. Mann, Edward Sambrano, Sanjay B. Maggirwar, Natalia Soriano-Sarabia

**Affiliations:** Department of Microbiology, Immunology and Tropical Medicine, George Washington University, Washington, DC, United States

**Keywords:** γδ T cells, HIV latency, immunotherapy, innate immnuity, allogeneic T cell

## Abstract

The major barrier to HIV cure is a population of long-lived cells that harbor latent but replication-competent virus, are not eliminated by antiretroviral therapy (ART), and remain indistinguishable from uninfected cells. However, ART does not cure HIV infection, side effects to treatment still occur, and the steady global rate of new infections makes finding a sustained ART-free HIV remission or cure for HIV-seropositive individuals urgently needed. Approaches aimed to cure HIV are mostly based on the “shock and kill” method that entails the use of a drug compound to reactivate latent virus paired together with strategies to boost or supplement the existing immune system to clear reactivated latently infected cells. Traditionally, these strategies have utilized CD8+ cytotoxic lymphocytes (CTL) but have been met with a number of challenges. Enhancing innate immune cell populations, such as γδ T cells, may provide an alternative route to HIV cure. γδ T cells possess anti-viral and cytotoxic capabilities that have been shown to directly inhibit HIV infection and specifically eliminate reactivated, latently infected cells *in vitro*. Most notably, their access to immune privileged anatomical sites and MHC-independent antigen recognition may circumvent many of the challenges facing CTL-based strategies. In this review, we discuss the role of γδ T cells in normal immunity and HIV infection as well as their current use in strategies to treat cancer. We present this information as means to speculate about the utilization of γδ T cells for HIV cure strategies and highlight some of the fundamental gaps in knowledge that require investigation.

## Introduction

### Therapeutic Strategies Targeting Chronic HIV Infection

The road to developing a complete cure for HIV is fraught with potholes and dead ends. Clearing cellular and anatomical reservoirs provides a unique set of challenges. Latent virus is able to evade immune responses by integrating into the host genome of resting CD4+ T cells and entering a state of dormancy. Despite the cessation of new virion production, viral persistence is maintained by clonal expansion of HIV-infected cells (Chomont et al., 2009; Lee et al., 2020). Additional barriers include immune privileged or hard to reach anatomical sites such as the central nervous system, intestines, or secondary lymphoid organs where the virus can persist in the presence of Antiretroviral Therapy (ART) (Barton et al., 2016; Bronnimann et al., 2018; Denton et al., 2019; McManus et al., 2019). To date, HIV cure has only been achieved in two HIV-seropositive individuals who also had either acute myeloid leukemia or Hodgkin's lymphoma. Both individuals received allogeneic transplantation of stem cells carrying a homozygous mutation within the CCR5 gene (CCR5Δ32/Δ32), a chemokine receptor that facilitates viral entry (Gero Hütter et al., 2009; Gupta et al., 2019). However, it is unlikely that these practices may be feasible for wide-spread implementation. A safer, potentially more practical approach, called “shock and kill” has been the primary focus of cure research for the past 15 years (Sengupta and Siliciano, 2018). This strategy is based on the use of drug compounds, or latency reversing agents (LRAs), to reactivate viral replication followed by a treatment to enhance immune responses capable of eliminating reactivated latently HIV-infected cells (Deeks, 2012; Barton et al., 2013; Archin et al., 2014). A recent review by Kim et al. visits current LRAs under different stages of investigation (Kim et al., 2018). LRAs are classified into groups based on their primary intracellular targets. Epigenetic modifiers include histone deacetylase inhibitors (HDACi), histone methyltransferase inhibitors (HMTi), DNA methyltransferase inhibitors (DNMTi), bromodomain inhibitors (BRDi), and protein kinase C (PKC) agonists (Margolis et al., 2016). Non-epigenetic LRAs include agonists for the endosomal pattern recognition receptors TLR7, TLR8, and TLR9, which have been shown to increase both viral transcription as well as anti-HIV innate immune responses (Offersen et al., 2016; Lim et al., 2018; Meas et al., 2020). Unfortunately, within these categories, only a handful of drugs have progressed to animal studies or human clinical trials. These include the HDACis vorinostat, panobinostat, and romidepsin, the PI3K/Akt inhibitor disulfiram, PKC agonists bryostatin and ingenol, and the TLR9 agonist MGN1703. Excluding bryostatin, each of these compounds prompted an increase in detectable viral mRNA although this was not accompanied by clearance of infected cells (Kim et al., 2018). Therefore, the viability of the “shock and kill” strategy is contingent upon the discovery and development of novel LRAs with different mechanisms of action and possibly target alternative pathways.

The majority of current LRAs reactivate viral transcription through induction of the canonical NF-κB pathway. NF-κB is a host transcription factor that interacts with the HIV LTR and has been shown to be a powerful driver of the viral replication cycle (Nabel and Baltimore, 1987; Hiscott et al., 2001). This pathway is not limited to infected cells and therefore off-target toxicity via systemic immune activation remains a concern (Bratland et al., 2011). An immunosuppressive effect of LRAs on different effector immune cell subsets has also been reported, posing an additional complication when attempting to reconstitute total immune response (Garrido et al., 2016; Walker-Sperling et al., 2016). Despite these concerns, combining LRAs has been shown to produce a synergistic effect and potentially abrogate uncontrolled T cell activation. Darcis et al. demonstrated an increase in efficacy across several *in vitro* and *ex vivo* latency models when either of the PKC agonists bryostatin or ingenol are paired with the bromodomain inhibitor JQ1. Building off of this work, Albert *et al*. found both an increase in efficacy and reduction in systemic activation when bryostatin is paired with HDACis (Darcis et al., 2015; Albert et al., 2017). In addition, modulation of the non-canonical NF-κB pathway through second mitochondrial-derived activator of caspases (SMAC) mimetics could potentially be of interest for shock and kill strategies (Pache et al., 2015). Induction of this pathway leads to more persistent NF-κB-driven transcription and thereby potentially avoids the detrimental side effects observed with previous LRAs. Most recently, the SMAC mimetic AZD5582 has been shown to induce robust HIV reactivation throughout the deep anatomical reservoirs of humanized mice and non-human primates. These studies, although extremely promising, need further evaluation and testing in humans (Sampey et al., 2018; Nixon et al., 2020). These developments in latency reversal should be paired with equally innovative immunotherapies to effectively target and clear chronic HIV infection.

### Cellular Based Immunotherapies for “Shock and Kill”

Harnessing the natural antiviral CTL immune response has been the most investigated strategy for the “shock and kill” approach (Borrow et al., 1994; Santra et al., 2010). This is highlighted by the development of HIV-specific *ex vivo* expanded T cells (HXTCs) capable of recognizing a variety of viral epitopes. HXTCs were shown to be safe for adoptive transfer into humans but had little effect on viral clearance in the absence of reactivation (Sung et al., 2018). Unfortunately, some LRAs including HDACis and PKC agonists may have deleterious effects on CTL function that requires further investigation (Clutton and Jones, 2018). The extent of these effects occurring *in vivo* and amongst other classes of LRAs is the subject of current clinical studies. Furthermore, CTL-based strategies continue to struggle with issues stemming from viral escape, immune exhaustion, and inaccessibility to anatomical reservoirs, including the B cell follicle (Day et al., 2006; Connick et al., 2007; Deng et al., 2015). Alternative strategies that utilize NK cells are starting to be explored, and their potential as immunotherapy in HIV infection has recently been reviewed (Desimio et al., 2019).

Additionally, the use of γδ T cells could offer a novel therapeutic avenue that may overcome some of the challenges facing traditional αβ T cell strategies. γδ T cells possess a range of antiviral function including cytolytic activity against HIV-infected cells (Wallace et al., 1996). Specifically, our group showed that Vδ2 T cells from ART-suppressed HIV-infected individuals target and kill reactivated autologous HIV-infected CD4+ T cells *in vitro*, establishing the first proof of concept of the capacity of Vδ2 T cells to be used in immunotherapeutic approaches toward an HIV cure (Garrido et al., 2018). Further proof comes from our recent work showing a correlation between γδ T cell cytotoxic capacity with a lower recovery of replication-competent HIV in cultures of resting CD4+ T cells from ART-suppressed HIV-seropositive individuals (James et al., 2020). In addition, activated γδ T cells induce adjuvant immune responses including HIV-specific T cell responses (Poccia et al., 2009). The clinical utilization of γδ T cells for HIV cure remains relatively understudied compared to the cancer field, but these initial findings paired with an examination of their basic biology warrants further investigation into their potential as an immunotherapy.

## γδ T Cells: A Critical Bridge Between Innate and Adaptive Immunity

### Characteristics of γδ T Cells

The γδ T cell lineage is a unique subset of innate-like T lymphocytes that offer an attractive alternative to conventional αβ T cells, which predominate in current cell-based immunotherapies. Since their discovery in the 1980's, γδ T cells have been shown to contribute to tumor surveillance, fighting infectious disease, and autoimmunity (Tanaka, 2006; Kabelitz, 2011; Vantourout and Hayday, 2013; Silva-Santos et al., 2015; Lawand et al., 2017). Their defining feature is a T-cell receptor (TCR) comprised of variable γ and δ chains that recognizes non-peptidic antigens in the absence of Major Histocompatibility Complex (MHC) molecules (Brenner et al., 1986; Holoshitz et al., 1993; Tanaka et al., 1995). γδ T cells develop and mature in the thymus and constitute the first T cell population to migrate and populate the periphery during fetal development. Interestingly, there is a correlation between tissue localization and the Vδ chain expression in the TCR indicating a predefined role for γδ T cells before leaving the thymus (Zhao et al., 2018). Although the specific mechanisms of γδ T cell development and differentiation are still being elucidated, the correlation between Vδ chain expression and tissue localization is well-established (Munoz-Ruiz et al., 2017).

Human γδ T cells account for 0.5–10% of circulating T cells and are classified into two major subpopulations based on the δ chain usage, Vδ1 and Vδ2 T cells. Smaller subpopulations expressing Vδ3 and Vδ5 share a degree of similar functions as Vδ1 T cells but the extent of their involvement in immunity is unknown (Takihara et al., 1989; Halary et al., 2005). The Vδ2 subpopulation is almost always paired with the Vγ9 chain (Vγ9Vδ2) and while the Vδ1 subpopulation is also capable of pairing with Vγ9, it is less frequently observed (Dimova et al., 2015). Vδ2 represents the majority of γδ T cells found in the peripheral blood whereas the Vδ1 subpopulation primarily resides in tissues such as the gut mucosa, lungs, and female reproductive system (Itohara et al., 1990; Wu et al., 2017).

Vδ1 T cells are capable of recognizing self-antigen lipids presented within the MHC-like CD1 protein family, but the specific ligand they recognize remains unknown. In contrast, Vδ2 T cell ligand has been extensively characterized. Vδ2 T cells undergo activation following recognition of low-molecular-weight phosphorylated compounds referred to as phosphoantigens (P-Ags) (Jomaa et al., 1999; Green et al., 2004; Adams et al., 2015). These P-Ags are metabolic intermediates of the isoprenoid biosynthesis pathway. Isoprenoids are a diverse class of organic compounds involved in a number of biological processes ranging from cell membrane maintenance to protein regulation. As such, isoprenoids are produced both endogenously as well as by invading microbes. The most potent, naturally occurring P-Ag commonly found in pathogenic bacteria is (E)-4-Hydroxy-3-methyl-but-2-enyl pyrophosphate (HMB-PP), an intermediate of the MEP (non-mevalonate) pathway (Hintz et al., 2001). A second, less potent activator found in eukaryotic cells is Isopentenyl pyrophosphate (IPP), an intermediate of the mevalonate pathway that accumulates during periods of cellular stress (Gossman and Oldfield, 2002; Gober et al., 2003). Although γδ T cells were discovered more than 30 years ago, our understanding of how Vδ2 cells recognize P-Ag has been a slow process. Early on, it was suggested that presentation occurred outside of conventional MHC or known MHC-like molecules but still required cell to cell contact (Morita et al., 1995). It was only recently discovered that the ubiquitously expressed B7 family protein, CD277 (butyrophilin-3/BTN3A), acts as a mediator of P-Ag presentation to Vδ2 cells (Harly et al., 2012; Rhodes et al., 2015; Sebestyen et al., 2016; Yang et al., 2019). Furthermore, the specific antigens recognized by other γδ T cell populations remain elusive. In addition, it is unclear if BTN3A is the sole protein directly involved in presenting PAgs or if it is part of a larger mechanism involving a number of intracellular proteins or membrane bound transporters.

γδ T cells are further characterized by a diverse set of membrane bound receptors and cytokine production capability that denotes a high degree of polyfunctionality as regulatory and effector cells (Vantourout and Hayday, 2013). γδ T cells shape the total immune response by secreting a number of regulatory cytokines and interacting directly with other immune cell populations (Wu et al., 2014). Distinct subpopulations are capable of producing anti-inflammatory or pro-inflammatory signals and play a direct role in immune regulation in normal health and disease (Kuhl et al., 2009; Wakita et al., 2010; Ma et al., 2011; Coffelt et al., 2015; Peters et al., 2018). The expansive role of γδ T cells as immune modulators is underscored by their interactions with other immune cell populations. Some of these interactions are reciprocal in nature; DCs improve the antigen specific response of γδ T cells which in turn facilitate DC maturation (Conti et al., 2005; Martino et al., 2005). Activated peripheral γδ T cells can either induce anti-tumor activity of NK cells through CD137L (4-1BBL) expression or mediate NK pro-inflammatory cytokine production and DC editing through CD278 (ICOS) co-stimulation (Maniar et al., 2010; Nussbaumer et al., 2011; Cairo et al., 2014). Interestingly, activated peripheral γδ T cells also possess professional antigen-presenting cell (APC) capabilities that enable them activate naïve CD8+ αβ T cells and thus stimulate the development and differentiation of the CTL response against pathogens and tumors (Brandes et al., 2009; Khan et al., 2014).

### γδ T Cells Are Potent Cytotoxic Effectors

Most γδ T cells express the NK cell receptors, DNAM-1 and NKG2D, the latter of which acts as a co-stimulatory signal upon recognition of stress markers MICA, MICB, and ULBPs. Activation through this pathway has a 2-fold effect resulting in the secretion of pro-inflammatory cytokines TNF-α and IFN-γ as well as direct cytolytic activity mediated by the release of perforins and granzymes (Wu et al., 2002; Rincon-Orozco et al., 2005; Gonzalez et al., 2008; Toutirais et al., 2009). In addition, γδ T cells can induce apoptosis through the expression of TNF-related apoptosis-inducing ligand (TRAIL) or FasL, highlighting their role in clearing tumor cells as well as activated immune cells during the resolution of inflammation (Dalton et al., 2004; Ponomarev and Dittel, 2005; Todaro et al., 2009). Effector γδ T cell subsets can express CD16 (FcγRIII), affording them the ability to take part in ADCC of virally-infected cells as well as opsonization-aided phagocytosis of cell free pathogens (Chen and Freedman, 2008; Wu et al., 2009). As part of the first line of defense, they readily express toll-like receptors (TLR) that allow recognition and response to various microbial pathogens that invade barrier tissues (Wesch et al., 2011). Although γδ T cells exhibit many functions ascribed to innate immunity, they have a memory phenotype that can be defined by CD45RA/CD27/CD28 and CCR7 expression. This is consistent with other lymphocytes within the adaptive compartment, but collectively γδ T cells exert non-redundant functions (Dieli et al., 2003; Pitard et al., 2008; Ryan et al., 2016; Guerra-Maupome et al., 2019). Defining the complete mechanisms by which γδ T cells recognize foreign and self-ligands will require thorough investigation. Elucidating these mechanisms will further improve our understanding of γδ T cell biology as well as enable us to design efficacious therapeutic strategies. This is especially evident in the context of HIV as we currently lack a comprehensive understanding of the role of γδ T cells during HIV infection and how they recognize virally infected cells.

### The Impact of HIV Infection and Antiretroviral Therapy on γδ T Cells

When assessing γδ T cells' prospect as a novel HIV cure therapeutic, it is crucial to understand the effects of both HIV pathogenesis and ART on γδ T cell populations. Pauza et al. have recently reviewed the initial studies of γδ T cell dysregulation in the context of HIV disease progression and treatment (Pauza et al., 2014). Here, we summarize those early findings in addition to key details uncovered by more recent work. The most striking effect of primary HIV infection on γδ T cells is the inversion of the frequency of Vδ2:Vδ1 subpopulations within the peripheral blood. This event occurs early on, prior to the inversion of CD4:CD8 αβ T cells, and is driven by an expansion of Vδ1 T cells and depletion of Vδ2 T cells (Autran et al., 1989; Li et al., 2014). This difference in subpopulation outcomes can be partially explained by a few subtle yet important distinctions in the receptors they express. Like most gut-associated lymphocytes, both Vδ1 and Vδ2 T cells express the integrin α4β7, but only Vδ2 T cells express the chemokine receptor CCR5. Binding of the HIV envelope glycoprotein gp120 to α4β7 and CCR5 induces cell death (Li and Pauza, 2011, 2012). In addition, although γδ T cells are typically double negative (CD4-CD8-), Vδ2 T cell activation leads to a transient upregulation of the CD4 receptor (Lusso et al., 1995; Soriano-Sarabia et al., 2015). Furthermore, *ex vivo* phenotypic analysis of CD4 and CCR5 expression on viremic individuals in the acute phase of the infection revealed a transient increase in the expression of these receptors rendering Vδ2 T cells susceptible to entry by CCR5-tropic viruses (Soriano-Sarabia et al., 2015). Typically, only a small subset of peripheral Vδ2 T cells expresses the chemokine receptor CXCR4, but an increase in expression found in individuals with chronic infection raises the possibility that Vδ2 T cell may become susceptible to CXCR4-tropic viruses after initial infection (Imlach et al., 2003). Recovery of replication-competent virus from Vδ2 T cells confirmed the possibility of direct infection, but due to their low representation within total T lymphocytes it is difficult to quantify their contribution to the viral reservoir (James et al., 2020). The surviving Vδ2 T cell population shows attenuated responsiveness to P-Ag and effector functions. Consequently, Vδ2 T cells from HIV-seropositive individuals show diminished response to *in vitro* stimulation with IPP, reduced tumor recognition, as well as a significant loss of IFN-γ and TNF-α production (Wallace et al., 1997). It remains unclear if integrated provirus plays a role in these observed defects. While γδ T cell dysfunction begins early in primary HIV infection, comparative differences in the distribution of effector phenotype and function observed during acute vs. chronic infection indicate a dynamic interplay between γδ T cells and disease progression (Kosub et al., 2008; Cimini et al., 2015). On this note, it is critical to evaluate whether ART is able to reconstitute γδ T cell numbers and functionality at each stage of infection (Juno and Eriksson, 2019).

Although the Vδ2:Vδ1 inverted frequencies are never restored, early initiation of ART has been shown to partially restore the loss of γδ T cell function in HIV-seropositive individuals. Casetti et al. found that introducing treatment during primary infection reconstitutes Vδ1 T cell direct cytotoxic capabilities but antiviral chemokine production of CCL4 (MIP-1β) remains dampened despite early intervention. Moreover, both Vδ2 T cell cytotoxic function and pro-inflammatory cytokine production appear to be negatively impacted early on and are unable to be recovered regardless of the timing of ART (Casetti et al., 2019). Interestingly, our study in HIV-seropositive individuals on suppressive ART for more than 1 year showed that the remaining Vδ2 T cells retained their ability to degranulate in the presence of reactivated latently infected CD4+ T cells (Garrido et al., 2018). Whether or not ART is able to restore antigen responsiveness and polyfunctionality is being studied further within our group. Finally, the positive correlation between CD4+ T cell count and Vδ2 T cell quality in ART-suppressed HIV-seropositive individuals underscores the potential requirement of other immune cell populations needed to maintain or reconstitute γδ T cell function after primary HIV infection (Li et al., 2008; Casetti et al., 2015).

## Exploring the Potential of Allogeneic γδ T Cell Immunotherapy for HIV Cure

### Lessons Learned From the Treatment of Cancer

**Figure 1:**
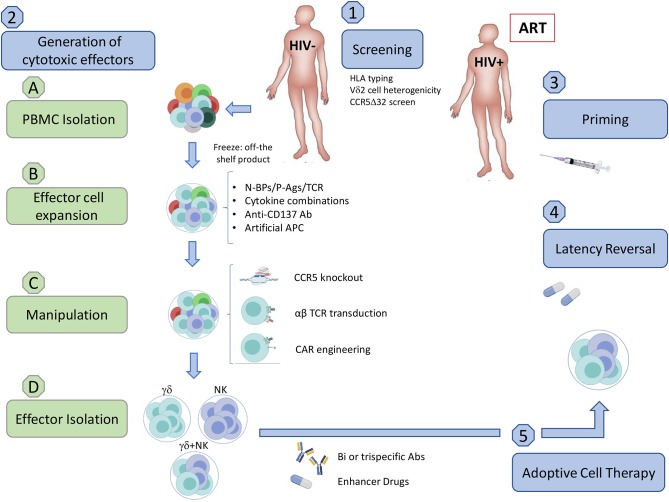
Implementation of an allogeneic adoptive cell therapy. (1) **Pre-clinical screening**. (A) Assess donor HLA compatibility. Although allogeneic γδ T cells should not generate GVHD and were reported to be safe in some clinical trials in cancer, we need to exercise caution until further clinical testing demonstrates the safety of allogenic transfer in ART-suppressed HIV-seropositive individuals. (B) Assess Vδ2 T cell population heterogenicity (C) Ideally use a CCR5Δ32/Δ32 donor. (2) **Generation of cytotoxic effector cells from uninfected donors** (A) PBMC Isolation from uninfected donors. (a) Density gradient separation from leukapheresis product from uninfected individuals. (b) Freeze PBMC to have an off-the shelf/ready to use product. (B) Expansion. (a) Expansion of Vδ2 T cells using a cocktail of optimized cytokines and activating compounds such as N-BPs, P-Ags, or TCR crosslinkers. Addition of feeder cells can increase the number and effector function of expanded cells. These may include artificial APCs (aAPC) expressing CD40L and CMV antigen pp65 for γδ T cells, or the K562 cell line for both γδ T cells and NK cells. (b) The natural crosstalk between γδ T cells and NK cells could be exploited via co-culturing to induce binding of CD137-CD137L resulting in a boost to NK cell cytotoxic function. Co-culture may be feasible with the addition of anti-CD137 Abs and select cytokine combinations that are beneficial for both cell types, such as IL-12 and IL-15. (C) Manipulation (a) Gene-editing enzymes such as zinc-finger nucleases or CRISPR-Cas9 could be utilized to generate γδ T cell effector subsets with a CCR5-knockout, reducing the likelihood of HIV infection or CCR5-mediated cell death. (b) Transduction of αβ TCR or CAR can be included in this step to boost specificity for peptidic HIV antigens expressed on the surface of infected cells (Figure 2). (A) Effector cell isolation/enrichment. (a) Cell sorting of either γδ T cell or NK cell populations. (b) Enrichment of γδ cells and NK cells (c) αβ T cell depletion. While multiple methods efficiently isolate effector populations, enrichment and αβ T cell depletion carry a higher risk for inducing GVHD. (d) Ready to use product. (A) **Priming**. Vaccination may boost pre-existing anti-HIV specific responses prior to adoptive transfer. (B) **Administering Latency Reversal Agents**. Reactivate latent virus with clinically approved HDACis, PI3K/Akt inhibitor, BRDi, or TLR agonist. (C) **Adoptive cell transfer**. (a) Allogeneic adoptive cell transfer of potent cytotoxic effectors. (b) Specific killing of HIV-infected cells could be achieved through the use of bi/trispecific antibodies that would recognize CD16 on one arm, γδ TCR on the other, and HIV proteins in the last arm of the antibody. (c) Enhancer drugs like bortezomib or the use of LRAs such as BRDis may increase *in vivo* efficacy by upregulating cytotoxic receptors on effector cells or their ligands on target cell populations. Created with BioRender.com.

In addition to altering γδ T cell frequencies, HIV infection modulates the expression of NK cell-like and potentially other receptors, directly modifying their cytotoxic capabilities (Poccia et al., 1999; Fausther-Bovendo et al., 2008; Hudspeth et al., 2012; Omi et al., 2014). We are currently missing a compelling picture of how HIV is recognized by γδ T cells, characterization of the various known receptors and mechanisms utilized to combat HIV infection reveals distinct similarities and differences between the two major γδ T cell subpopulations. Although both subpopulations display anti-HIV capabilities, investigations and clinical trials have been mostly based on Vδ2 T cells. Nevertheless, Vδ1 T cells have shown potential as an immunotherapy for cancer but lacking knowledge of the specific activating ligand poses an additional barrier (Silva-Santos et al., 2019). A significant advantage of using γδ T cells for immunotherapy comes from our detailed understanding of how to manipulate the mevalonate pathway to produce isoprenoid intermediates that activate Vδ2 T cells. Aminobisphosphonates (N-BPs) are structurally similar to pyrophosphates and specifically activate Vδ2 T cells by inhibiting farnesyl pyrophosphate synthase, leading to an accumulation of isopentenyl pyrophosphate (van Beek et al., 1999). N-BPs such as zoledronate and pamidronate are used for the treatment of low bone density-associated diseases including osteoporosis and cancer (Kunzmann et al., 1999; Berenson, 2001). Clinical trials in different malignancies first demonstrated that N-BPs combined with IL-2 strongly stimulate Vδ2 T cell activation and proliferation *in vivo*. However, some studies revealed that prolonged exposure to N-BPs leads to increased anergic Vδ2 T cells and concerns over IL-2 toxicity required refined dosing regimens (Kunzmann et al., 2000; Sicard et al., 2005; Lang et al., 2011). This led toward an adoptive transfer approach where cells could be expanded and monitored under controlled *in vitro* conditions. Despite results from these clinical trials showing mixed benefits, overall these strategies are well-tolerated and safe (Sato et al., 2005). Given the safety of these trials, we speculate that allogeneic γδ T cells from uninfected individuals have the potential to be utilized for an HIV cure and provide an overview of the clinical application of such an approach (Figure 1).

### *Ex vivo* Manipulation of γδ T Cells

One of the advantages of using γδ T cells is that they can be easily manipulated *in vitro*. N-BPs and P-Ags in combination with IL-2 have been shown to be strong stimulators of Vδ2 T cell activation and proliferation in *ex vivo* human PBMCs (Kunzmann et al., 2000). Alternative methods that could be used to induce *in vitro* activation of both major human subpopulations rely on cross-linking activating surface receptors after treatment with lectin-based compounds such as concanavalin A or antibodies that target the γδ TCR or CD3 complex (Dokouhaki et al., 2010; Siegers et al., 2011; Zhou et al., 2012). γδ T cells are receptive to further possible modulation by treatment with combinations of different exogenous cytokines. IL-2 and IL-15 selectively activate resting human Vδ2 T cells in the presence of P-Ag, promoting expansion and an increase in TNF-α and IFN-γ production (Kjeldsen-Kragh et al., 1993; Garcia et al., 1998). These cytokines are compatible or in some cases have synergistic effects when combined with IL-12, IL-18, or IL-21 favoring development of an effector memory phenotype with enhanced cytolytic activity (Thedrez et al., 2009; Li et al., 2010; Domae et al., 2017). Optimizing combinations of activating compounds and cytokines constitutes one of the approaches to properly expand and maintain desired Vδ2 T cell effector phenotypes. Determining which effector subset is best suited for HIV cure strategies and optimizing an expansion protocol to generate these effectors is currently being explored by our lab. Although the activating ligand of Vδ1 T cells has yet to be characterized, successful *in vitro* expansion protocols have been published, and constitute another avenue worthy of investigation for HIV cure. Almeida et al. generated the so-called Delta one T (DOT) cells, which are Vδ1-enriched γδ T cells with overexpressed cytotoxic NK cell receptors following treatment of PBMCs from healthy human donors with an anti-CD3 antibody, IL-4 and IFN-γ. Within a mouse model, these cells effectively trafficked to malignant tissues and displayed potent anti-tumor activity (Almeida et al., 2016).

**Figure 2:**
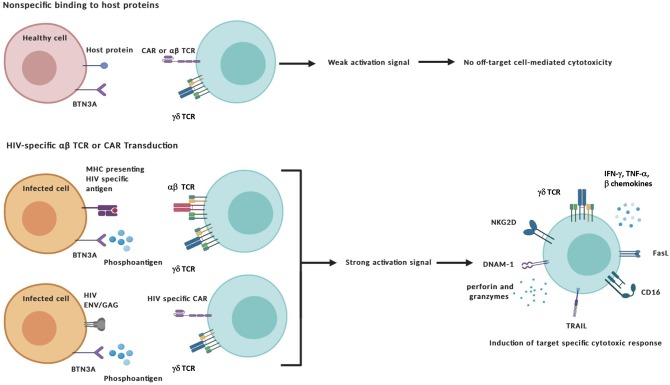
Specific targeting and activation of γδ T cell effectors. The transduction of HIV-specific αβ TCRs or chimeric antigen receptors (CAR) combined with the unique antigen recognition of γδ T cells could reduce off-target toxicity issues. Target-specific activation of γδ T cells can lead to the exertion of cytotoxic functions through a number of different mechanisms: (i) direct cytolysis of malignant or infected cells through the release of cytotoxic granules containing perforin and other granzymes; (ii) Induction of apoptosis by Fas Ligand and TRAIL death receptors; and (iii) antibody-dependent cellular cytotoxicity (ADCC) triggered by binding of the Fcγ receptor CD16 to the constant region of IgG antibody-coated targets. In addition to secreting pro-inflammatory cytokines interferon-γ (IFN-γ) and tumor-necrosis factor-α (TNF-α), γδ cells also produce CC-chemokines that inhibit CCR5-dependent entry of HIV virions into CD4+ T cells. Created with BioRender.com.

In addition to *ex vivo* expansion, γδ T cell therapies could stand to benefit from advances in gene editing or inclusion of immunomodulators such as designer antibodies or pro-cytotoxic drug compounds. Transduction of αβ TCRs could bestow γδ T cells with additional antigen recognition of intracellular peptides presented within MHC molecules whereas CARs could be engineered to recognize specific extracellular markers. Traditionally, these modifications have been applied to αβ T cells to target cancer with mixed results, the chief concern being off-target toxicity and ensuing “cytokine storm” that leads to systemic immune activation (van den Berg et al., 2015; Bonifant et al., 2016; Srivastava and Riddell, 2018). Applying these modifications to γδ T cells has shown promise for eliminating these issues while maintaining antigen-specific effector function and therefore constitute an additional advantageous manipulation to consider when targeting HIV-infected cells (Figure 2) (Harrer et al., 2017; Capsomidis et al., 2018).

**Figure 3:**
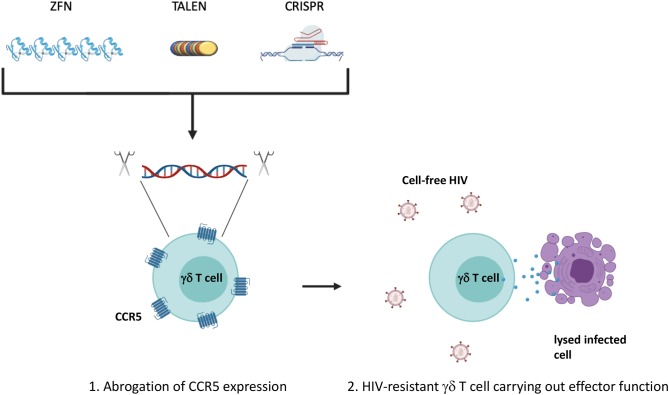
Manipulation of CCR5 expression. The expression of CCR5 leaves Vδ2 T cells susceptible to direct infection as well as activation of the p38-mediated cell death signaling pathway. (1) Ablation of the CCR5 gene could confer a degree of resistance to effector subsets prior to adoptive transfer generating (2) HIV-resistant γδ T cells. Traditional gene editing strategies have utilized zinc-finger nucleases or transcription activator-like effector nucleases (TALENs) to recognize and cleave specific genomic sequences. More recently, focus has shifted to the use of clustered regularly interspaced short palindromic repeats (CRISPR) and CRISPR associated protein 9 (Cas9) that rely on a small single guide RNA that is complementary to the target gene sequence. Created with BioRender.com.

Due to Vδ2 T cells susceptibility to CCR5-mediated cell death and direct infection, the allogeneic adoptive cell therapy would require either finding CCR5Δ32 homozygous healthy individuals or *in vitro* manipulation of the expanded Vδ2 T cells to abrogate CCR5 expression prior to administration into ART-suppressed HIV-seropositive individuals (Li and Pauza, 2011; Soriano-Sarabia et al., 2015). Since learning of the protective effect of the CCR5Δ32/Δ32 mutation, researchers have attempted to manipulate the expression of CCR5 through the use of gene-targeting nucleases (Haworth et al., 2017). Recent studies have shifted to the use of clustered regularly interspaced short palindromic sequences (CRISPR) and CRISPR associated protein 9 (Cas9) which have been shown to successfully confer resistance to transplanted hematopoietic stem cells within a mouse model as well as CD4+ T cells *in vitro* (Xu et al., 2017; Yu et al., 2018). Improvements in this area of research could lead to the development of γδ T cells or other adoptive cellular therapies resistant to infection by CCR5-tropic HIV (Figure 3). In addition, co-administration of bispecific or trispecific antibodies to facilitate the lysis of both cancer and HIV-infected cells with a high degree of specificity by binding to a target specific epitope on one arm, and an effector cell receptor, such as CD3 or CD16, on the other arm could also be applied for increased γδ T cell cytotoxic specificity (Ferrari et al., 2016; Schiller et al., 2016). Additionally, co-culturing γδ T cells with NK cells treated with an anti-CD137 Ab could promote their interaction *in vitro* leading to increased expansion and cytotoxic capability (Chu et al., 2019; Vidard et al., 2019). Further boosting of effector function could be achieved by drug-mediated upregulation of cytotoxic receptor ligands on the surface of target cells (Niu et al., 2017). Lessons from these cross-disciplinary studies could be applied to increase the efficacy of strategies utilizing γδ T cells to treat persistent HIV infection. Taken together, γδ T cells are amenable to a variety of *in vitro* modifications that could be optimized for generating tailored cytotoxic effector populations.

## The Approach: Allogeneic γδ T Cell Immunotherapy

Although the anti-HIV capacity of γδ cells to inhibit viral replication is well-documented (Wallace et al., 1996; Soriano-Sarabia et al., 2015), only recently we have reported the proof of concept of their capacity to specifically kill reactivated latently infected cells (Garrido et al., 2018). This work sets the basis for further investigation toward understanding mechanisms of HIV recognition and specific killing toward an HIV cure. We speculate that an allogeneic strategy that utilizes prescreening of uninfected donors for the CCR5Δ32/Δ32 mutation or gene editing of CCR5 as the only possible way to implement the adoptive transfer of γδ T cells for HIV cure since γδ T cells can be infected by HIV (Poccia et al., 1999; Imlach et al., 2003; Soriano-Sarabia et al., 2015), and their contribution in the tissues, where they are numerically more abundant, remains unknown.

The study of allogeneic γδ T cell transfer to treat chronic disease is still in its infancy but there have been recent developments in the cancer field. Haploidentical hematopoietic stem cell transplantation markedly improved survival rates of patients with hematologic cancers (Godder et al., 2007). Heightened, post-transplantation levels of γδ T cells were determined to be a critical component of the trial's success. The key findings of this study revealed γδ T cells could achieve strong anti-leukemic activity in the absence of graft-vs.-host disease. A follow up study by Wilhelm *et al*. reinforced this observation by transplanting haploidentical γδ T cells and expanding them *in vivo*. With three out of four patients experiencing brief but complete remission and no major side effects, allogeneic transfer shows potential as an immunotherapeutic tool (Wilhelm et al., 2014). Most recently, a regimen of allogeneic *ex vivo* expanded γδ T cells was used in the treatment of an individual with late-stage cholangiocarcinoma. In addition to anti-tumor activity, the authors observed signs of positive immune regulation in autologous αβ T cell and NK cell populations that possibly contributed to the improved clinical outcome (Alnaggar et al., 2019). This suggests that the transfer of γδ T cells may act directly against malignant tissue as well as recruit other immune populations to join in the anti-tumor response. It should be noted that each of these cases include patients that may have been immunosuppressed or exposed to a myriad of previous treatments that could exert an impact on these findings.

These studies provide the possibility that allogeneic transfer may be a safe, feasible treatment for certain cancers. Whether this applies to relatively healthier ART-treated individuals will require direct studies to sufficiently assess safety and efficacy for HIV cure. Nevertheless, as a byproduct of the cancer trials, untethering from strict HLA-matching engenders the possibility of generating an off-the-shelf repository of γδ T cells to treat disease. Improvements to *ex vivo* expansion protocols as a prior step toward generating a powerful cytotoxic γδ T cell population, would bring this prospect closer to reality. Several groups have shown that automation of cell isolation and co-culture with artificial APCs expressing co-stimulatory molecules such as CD40L and CMV antigen pp65 can drastically increase the number, purity, and effector function of the expanded γδ T cells while maintaining compliance with current good manufacturing practices (Lamb et al., 2018; Polito et al., 2019). Despite these improvements, the inherent heterogeneity of γδ T cell populations between potential donors could pose a significant challenge to manufacturing a consistent cellular product barring the development of an efficient pre-clinical screening method (Cairo et al., 2010; Ryan et al., 2016). Although many basic science questions will need adequate attention, allogeneic transfer presents an enticing strategy for utilizing γδ T cells as a multipurpose immunotherapy.

## Further Considerations

### Effect of LRAs on Immune Cell Function

It is important to determine how these compounds impact the cytotoxic capacity of effector cells in HIV-seropositive individuals. Epigenetic modifiers, belonging to the same categories as current LRAs, have been shown to affect γδ T cell or NK cell functions. Current information about the impact of HDACis on γδ T cells is limited and comes from cancer studies using Valproic acid (Bhat et al., 2019), which was shown to be ineffective at reactivating persistent HIV *in vivo* (Archin et al., 2008). This cancer study showed a downregulation of the NKG2D receptor and redistribution of γδ T cell memory subsets (Bhat et al., 2019). Additional studies have focused on the effect of different LRAs on NK cell function showing mixed results. For example, panobinostat (PNB) had deleterious effects to NK function by reducing cytotoxicity and IFN-γ production (Rasmussen et al., 2013; Garrido et al., 2016). However later *in vivo* observations did not support *in vitro* findings, highlighting the absolute requirement of *in vivo* validation (Garrido et al., 2019). Furthermore, the PKC agonist prostatin (PROST) may have beneficial impact on NK function by increasing NKG2D expression (Garrido et al., 2016). Conversely, bromodomain inhibitors have been shown to increase NKG2D ligands on the surface of cancer cells with the added benefit of downregulating PD-L1, potentially boosting cytotoxicity while mitigating immune exhaustion (Abruzzese et al., 2016; Zhu et al., 2016). These studies highlight the need to characterize the cellular effects of LRAs and strategically choose LRA types, doses, and administration regimens that optimize latency reversal without negatively impacting the antiviral activity of effector cells.

### Potential Use of γδ T Cells for HIV Cure

A number of fundamental questions that require investigation will need to be answered prior to the clinical implementation of γδ T cells in HIV therapy. How do γδ T cells recognize HIV infected cells? Do allogeneic γδ T cells specifically target and kill reactivated latently infected cells similar to autologous cells? Will the Vδ2 subpopulation alone be sufficient or will both subpopulations be required to maximize efficacy of an allogeneic approach? Additionally, what is the ideal cytotoxic phenotype for clearing reactivated latently infected cells and how will this phenotype be produced through *ex vivo* manipulation? Will the heterogeneity between donors preclude scaling up production and implementation? Our initial study only compared the expansion of Vδ2 T cells using PAM + IL-2 to cells expanded with HMB-PP + IL-2 and IL-2 alone (Garrido et al., 2018). Previous studies have shown the positive effects of IL-12, IL-15, IL-18, and IL-21 on producing *ex vivo* expanded Vδ2 T cell cytotoxic phenotypes (Thedrez et al., 2009; Van Acker et al., 2016; Domae et al., 2017). Similarly, we are currently optimizing Vδ2 T cell expansion to consistently produce a highly homogeneous cytotoxic phenotype despite the interindividual variation of Vδ2 T cell populations (Ryan et al., 2016). A *post hoc* comparison of donor phenotypes prior to expansion may also help identify the subset receptor expression profiles most likely to produce our desired cytotoxic effectors. Identification of the activation, cytotoxic, or chemotactic markers expressed in the donors from this comparison study could help us develop a preclinical screening panel for potential future donors.

## Concluding Remarks

Targeted therapy utilizing the potent cytotoxic capabilities of γδ T cells is a compelling avenue of research that has broad implications in treating a variety of diseases. Extensive focus within the field of cancer continues to produce a steady stream of innovations that may boost the efficacy of γδ T cell therapies. Advances in latency reversal combined with the allogeneic transfer of expanded effector γδ T cells could provide a dynamic two-prong strategy to cure persistent HIV infection. While our recent work showed the feasibility of using γδ T cells to specifically target and kill HIV infected cells (Garrido et al., 2018), a more detailed investigation into γδ T cell responses to HIV infection, and specifically persistent HIV infection, is needed before moving into clinical application. Based on the current knowledge summarized in this review, γδ T cells merit further consideration in investigations toward an HIV cure. Allogeneic transfer of *ex vivo* expanded γδ T cells presents an intriguing therapeutic option that may 1 day bring us a step closer to a sustained ART-free HIV remission or complete cure.

## Author Contributions

All authors have contributed to writing, editing, and reviewing the manuscript.

## Conflict of Interest

The authors declare that the research was conducted in the absence of any commercial or financial relationships that could be construed as a potential conflict of interest.
